# Land masses and oceanic currents drive population structure of *Heritiera littoralis*, a widespread mangrove in the Indo‐West Pacific

**DOI:** 10.1002/ece3.6460

**Published:** 2020-06-03

**Authors:** Achyut Kumar Banerjee, Wuxia Guo, Sitan Qiao, Weixi Li, Fen Xing, Yuting Lin, Zhuangwei Hou, Sen Li, Ying Liu, Yelin Huang

**Affiliations:** ^1^ State Key Laboratory of Biocontrol and Guangdong Provincial Key Laboratory of Plant Resources School of Life Sciences Sun Yat‐sen University Guangzhou Guangdong China; ^2^ South China Botanical Garden Chinese Academy of Sciences Guangzhou Guangdong China; ^3^ Division of Ecology & Biodiversity School of Biological Sciences The University of Hong Kong Hong Kong China

**Keywords:** chloroplast DNA, divergence, glacial refugia, phylogeography, Pleistocene, vicariance

## Abstract

Phylogeographic forces driving evolution of sea‐dispersed plants are often influenced by regional and species characteristics, although not yet deciphered at a large spatial scale for many taxa like the mangrove species *Heritiera littoralis*. This study aimed to assess geographic distribution of genetic variation of this widespread mangrove in the Indo‐West Pacific region and identify the phylogeographic factors influencing its present‐day distribution. Analysis of five chloroplast DNA fragments’ sequences from 37 populations revealed low genetic diversity at the population level and strong genetic structure of *H. littoralis* in this region. The estimated divergence times between the major genetic lineages indicated that glacial level changes during the Pleistocene epoch induced strong genetic differentiation across the Indian and Pacific Oceans. In comparison to the strong genetic break imposed by the Sunda Shelf toward splitting the lineages of the Indian and Pacific Oceans, the genetic differentiation between Indo‐Malesia and Australasia was not so prominent. Long‐distance dispersal ability of *H. littoralis* propagules helped the species to attain transoceanic distribution not only across South East Asia and Australia, but also across the Indian Ocean to East Africa. However, oceanic circulation pattern in the South China Sea was found to act as a barrier creating further intraoceanic genetic differentiation. Overall, phylogeographic analysis in this study revealed that glacial vicariance had profound influence on population differentiation in *H. littoralis* and caused low genetic diversity except for the refugia populations near the equator which might have persisted through glacial maxima. With increasing loss of suitable habitats due to anthropogenic activities, these findings therefore emphasize the urgent need for conservation actions for all populations throughout the distribution range of *H. littoralis*.

## Graphical abstract

This is the first report of the geographic distribution of genetic variation of a widespread mangrove (*Heritiera littoralis*) in the Indo‐West Pacific region. Being conducted at a large spatial scale, our study identified that natural geographic barriers, contemporary oceanic currents, and species ecology act interactively to shape present‐day distribution of the species in this region.

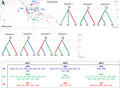

## 1 INTRODUCTION

Mangroves comprise of tropical and subtropical intertidal plant communities and are valuable for providing a range of essential ecosystem services, for example, sequestering carbon, protecting coastlines, and supporting coastal food webs (Barbier et al., 2011; Wee et al., 2019). Due to foreseeable future of mangroves extinction through human exploitation, climate change, natural geographic events, and cryptic ecological degradation (Dahdouh‐Guebas et al., 2005; Duke et al., 2007; Lohman et al., 2011), it is important to understand their evolutionary history and present distribution. Unlike terrestrial plants, most mangroves have buoyant propagules capable of long‐distance dispersal (LDD) through ocean currents for an extended period of time (Tomlinson, 2016), which plays a pivotal role in shaping population structure of mangroves by maintaining gene flow between geographically distant populations. Previous studies have identified that multiple factors including glacial vicariance and contemporary oceanic currents (e.g., Wee et al., 2015, 2017) have acted as barriers to propagule dispersal and thereby can influence the geographic distribution of genetic variation within the mangroves species.

However, influence of these phylogeographic factors driving population structure is not consistent across mangroves taxa and geographic regions. For example, land masses like Sunda (present day, the Malay Peninsula) and Sahul shelves, emerged during the glacial sea level changes, were found to cause genetic differentiation in many mangroves in the Indo‐West Pacific (IWP) region (e.g., Guo et al., 2016; Li et al., 2016). Recent phylogeographic studies, however, found evidence of genetic exchange across the land barriers, especially during interglacial periods of the Pleistocene when the land shelves resubmerged and provided corridors for genetic exchanges between the oceanic regions (Yang et al., 2017). The spatial pattern of genetic differentiation across these land barriers was also not consistent for all mangroves taxa. For example, *Bruguiera gymnorhiza* was found to be genetically differentiated across the Malay Peninsula (Minobe et al., 2010) whereas in case of *Rhizophora mucronata*, the genetic differentiation was found only at the edge of the Andaman Sea and the Strait of Malacca (Wee et al., 2014). Besides, LDD may become restricted by land and water (e.g., ocean currents) barriers, although the restrictions may vary across species depending on the mobility and survivability of the propagules (Duke, Lo, & Sun, 2002). In fact, the Indian Ocean has been found as an effective dispersal barrier only for those species having propagules with floating period less than six months (Van der Stocken, Carroll, Menemenlis, Simard, & Koedam, 2019). These findings indicate that phylogeographic forces may act at a finer scale at which regional—and species characteristics may interactively influence the genetic structure of mangroves populations. In this context, it is important to study the taxon‐specific genetic structure of major mangrove lineages through regional sampling design across their distribution range for a better understanding of the phylogeographic factors shaping the geographic distribution of genetic variation (Wee et al., 2017).

**Figure 1:**
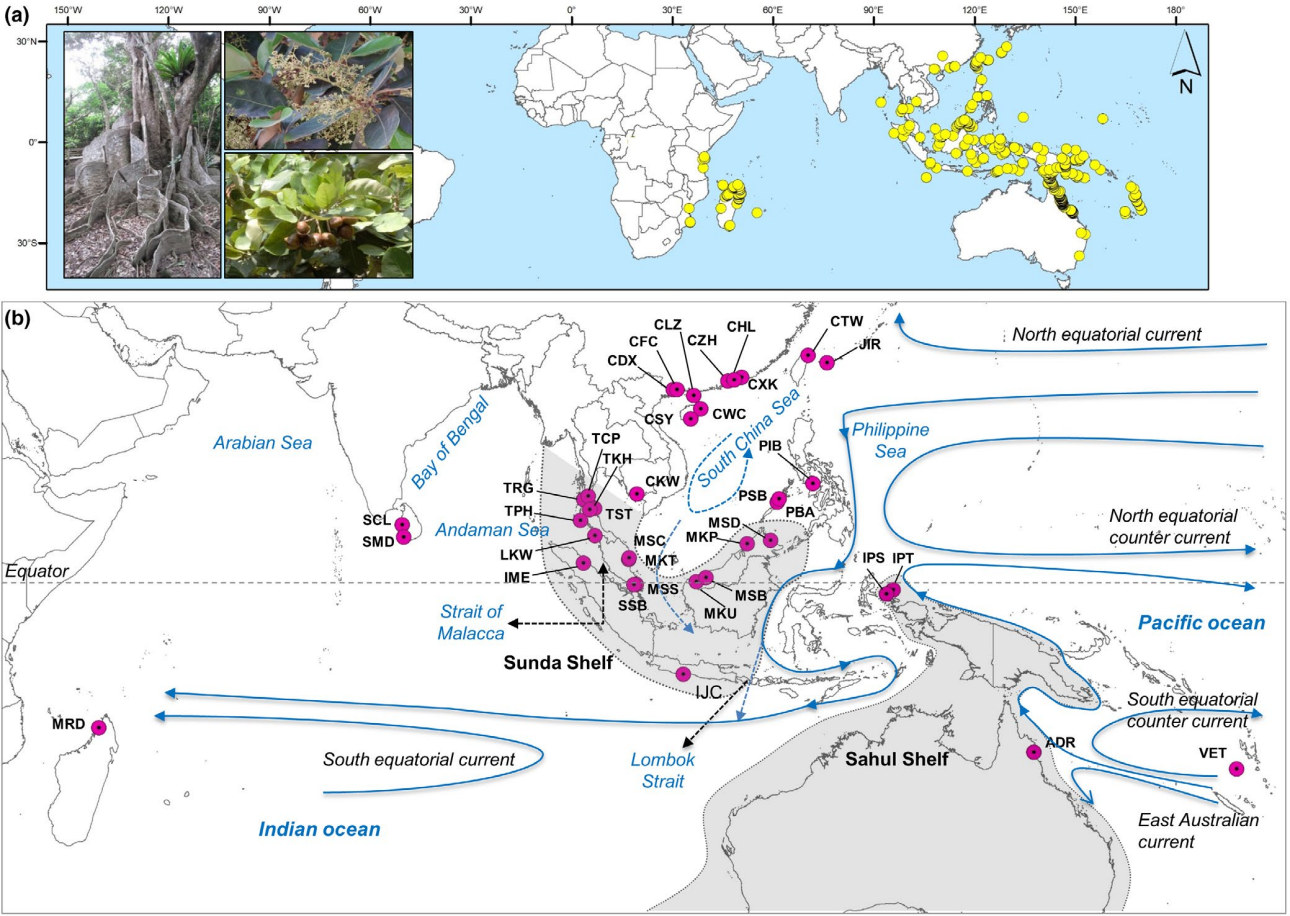
Distribution of *Heritiera littoralis* in the Indo‐West Pacific (IWP) based on—(a) occurrence records in the GBIF, and (b) actual sampling locations of this study. Abbreviations of the sampling sites have been given in Appendix S1. The locations of the Sunda and Sahul shelves, and major oceanic currents of the Indonesian Throughflow have been adopted from Lohman et al. (2011) and Hall (2009)

*Heritiera littoralis* Dryand. (Malvaceae), also known as the looking‐glass mangrove, occurs in the IWP region and is distributed from east Africa to southern Asia, Australia, and Melanesia (Figure 1). Based on physiological characteristics (e.g., salt tolerance and leaf traits), *H. littoralis* has been identified as a mangrove associate with lower salt tolerance than true mangroves and growing in the intertidal region and tidal estuaries, along the riverbanks, at the inward fringe of mangrove swamps and inland habitats (Jian, Tang, Zhong, & Shi, 2010; Wang, Mu, Li, Lin, & Wang, 2010). The fibrous mesocarp of *H. littoralis* propagules helps the species maintain a buoyancy period of several months (Van Der Stocken, Wee, et al., 2019) and this morphological adaptation provides the species with the LDD ability to attain widespread distribution in the IWP.

Previous studies have investigated the genetic structure of *H. littoralis* through dominant markers (RAPD, AFLP and ISSR) at a small spatial scale covering part of its distribution range (e.g., China, Japan, and Thailand) (Futai, Isagi, & Watanabe, 2010; Jian, Tang, Zhong, & Shi, 2004; Jian et al., 2010). These studies reported high level of genetic variation at population and species levels and high genetic differentiation among populations. Presence of prominent genetic structure of the species at small spatial scale was also found, probably driven by life history traits, LDD ability of floating seeds, and local environments. However, given the wide distribution of *H. littoralis* in the Palaeotropics, these studies lack the spatial resolution of the population structure. The relative importance of factors driving genetic structure (e.g., gene flow, environmental heterogeneity) could vary along the spatial scale (Anderson et al., 2010), and therefore, studies at large spatial scale are necessary for a comprehensive understanding of the influence of historical and contemporary genetic forces in shaping present‐day population structure. Furthermore, due to lack of quantitative measurement of genetic diversities from the presence–absence data matrix of amplified fragments (Triest, 2008), the phylogeographic inference of these studies was restricted. Maternally inherited chloroplast DNA (cpDNA) is especially informative in phylogeny of a species and allows inference of historical range shifts and recolonization routes (Bai, Liao, & Zhang, 2010). Recent studies have, therefore, used cpDNA to assess phylogeographic pattern in a number of mangroves (e.g., Guo, Guo, et al., 2018; Sun et al., 2019; Tomizawa et al., 2017), and similar accounts do not exist for *H. littoralis*.

This study was therefore conducted to examine the phylogeographic pattern of *H. littoralis* at a large spatial scale covering its nearly entire distribution range in the IWP. In line with what observed for many mangroves of this region, we hypothesized that LDD ability of propagules would explain its wide geographic distribution in the IWP and impediment of gene flow by the natural geographic barriers, such as the Sunda and Sahul shelves, or ocean current barriers such as the Indonesia‐Throughflow would generate prominent genetic structure of *H. littoralis* in this region. Specifically, we were interested in—(a) assessing the genetic diversity and population structure of the species across the geographic range, and (b) identifying the phylogeographic forces which would influence the present distribution of the species. We initially screened published primers to select five cpDNA loci having single nucleotide polymorphism (SNP) which were sequenced and analyzed further to infer the population genetic structure of *H. littoralis*.

## 2 MATERIALS AND METHODS

### 2.1 Sampling, DNA extraction, and PCR amplification

A total of 375 leaf samples of *H. littoralis* were collected from 37 locations across its distribution range (Figure 1; Appendix S1) with sample sizes varying from 4 to 15 individuals per sampling location (Table 1). To avoid the familial sampling bias, the individuals were selected maintaining a distance between at least 5 m. The leaf tissues were dried by silica gel and preserved in zip‐lock plastic pouches at −20°C until DNA extraction.

**Table 1:** Genetic diversity and historical demography parameters for each of the 37 sampling locations of *Heritiera littoralis* in the IWP

	Population	*N*	*H*	Hd	*π*	*V*	*S*	*P*	*T*‐*D*	*F*‐*F*s
POP I	CDX	5	1	0	0	NA	NA	NA	NA	NA
	CFC	11	1	0	0	NA	NA	NA	NA	NA
	CLZ	5	1	0	0	NA	NA	NA	NA	NA
	CWC	13	1	0	0	NA	NA	NA	NA	NA
	CZH	6	1	0	0	NA	NA	NA	NA	NA
	CXK	9	1	0	0	NA	NA	NA	NA	NA
	CHL	12	1	0	0	NA	NA	NA	NA	NA
	CSY	13	1	0	0	NA	NA	NA	NA	NA
	JIR	13	2	0.28 ± 0.14	0.22 ± 0.13	15	0	15	−0.52	1.11
	Total	87	2	0.05 ± 0.03	0.04 ± 0.02	15	0	15	**−2.17**	0.28
POP II	CTW	8	2	0.43 ± 0.17	0.34 ± 0.20	15	0	15	0.57	1.47
	PIB	6	3	0.73	0.33	12	1	11	1.09	1.44
	IPS	12	1	0	0	NA	NA	NA	NA	NA
	TCP	7	1	0	0	NA	NA	NA	NA	NA
	TPH	6	1	0	0	NA	NA	NA	NA	NA
	TKH	11	1	0	0	NA	NA	NA	NA	NA
	TST	10	2	0.47 ± 0.13	0.03 ± 0.02	1	0	1	0.82	0.90
	MKT	6	1	0	0	NA	NA	NA	NA	NA
	MSC	12	1	0	0	NA	NA	NA	NA	NA
	MSS	11	1	0	0	NA	NA	NA	NA	NA
	MKU	15	1	0	0	NA	NA	NA	NA	NA
	MSB	12	1	0	0	NA	NA	NA	NA	NA
	MKP	6	1	0	0	NA	NA	NA	NA	NA
	MSD	8	1	0	0	NA	NA	NA	NA	NA
	CKP	6	1	0	0	NA	NA	NA	NA	NA
	SSB	10	1	0	0	NA	NA	NA	NA	NA
	ADR	12	2	0.41 ± 0.13	0.04 ± 0.03	2	0	2	0.69	1.02
	MRD	11	1	0	0	NA	NA	NA	NA	NA
	Total	169	10	0.44 ± 0.05	0.057 ± 0.01	19	1	18	**−1.82**	0.04
POP III	VET	13	1	0	0	NA	NA	NA	NA	NA
	IJC	13	1	0	0	NA	NA	NA	NA	NA
	IPT	11	1	0	0	NA	NA	NA	NA	NA
	PBA	13	1	0	0	NA	NA	NA	NA	NA
	PSB	14	2	0.54 ± 0.05	0.23 ± 0.13	8	0	8	**2.69**	**1.96**
	Total	64	3	0.57 ± 0.04	0.14 ± 0.03	10	0	10	0.60	1.33
POP IV	IME	8	1	0	0	NA	NA	NA	NA	NA
	SCL	12	1	0	0	NA	NA	NA	NA	NA
	SMD	8	2	0.25 ± 0.18	0.01 ± 0.01	1	1	0	−1.05	−1.20
	TRG	12	1	0	0	NA	NA	NA	NA	NA
	LKW	15	1	0	0	NA	NA	NA	NA	NA
	Total	55	2	0.04 ± 0.03	0.002 ± 0.001	1	1	0	−1.09	−1.90
Overall	375	13	0.79 ± 0.01	0.34 ± 0.01	19	1	18		**3.07**	

Note

Grouping of samples under four populations (POP I‐IV) follows the spatial analysis of molecular variance (SAMOVA). Abbreviations of the sampling locations have been given in Appendix S1. Values reported are: number of individuals analyzed (*N*); number of haplotypes (*H*); haplotype diversity (Hd); nucleotide diversity (*π*); variable polymorphic sites (*V*), singleton variable sites (*S*); parsimony‐informative sites (*P*); standard deviation is for the sampling process for Hd and for both the sampling and the stochastic processes for *π*. *T*‐*D*, Tajima *D* test, *F*‐*F*s, Fu's *F*s test; significant values (*p* < .05) are in bold.

Total genomic DNA was extracted from dried leaf samples using the HiPure Plant DNA Mini Kit (Magen Technology Inc.) following the manufacturer's protocol. We initially screened 24 published primers for different cpDNA regions on a subset of three individuals per population randomly collected from six populations (i.e., MKP, CWC, SMD, LKW, JIR, VET) that were the farthest apart. Five fragments (i.e., *acc*D*‐psa*I,* trn*V‐*trn*M, *trn*S*‐trn*G,* atp*B*‐rbc*L,* rpl*16 spacer) that showed polymorphism within or among samples were finally chosen for the study (Appendix S2). DNA amplification for the 375 samples was carried out in a 30 μl reaction mix containing 1 µg of total DNA, 5 pmol of each primer, 10 mM of Tris–HCl (pH 8.4), 1.5 mM of MgCl_2_, 0.1 mM of dNTP, and 2 units of Taq polymerase (Shengong Inc.). The optimized thermal profile consisted of 5‐min initial denaturation step at 94°C, followed by amplification for 30 cycles of denaturing for 45 s at 94°C, annealing for 45 s at 53°C, extension for 1.5 min at 72°C, and a final cycle at 72°C for 10 min. Amplification products were inspected in 1% agarose gel with ethidium bromide and photographed under UV light. Purified DNA fragments were sequenced for both strands on an ABL 3730XL DNA Analyzer (Applied Biosystems Inc.).

### 2.2 Assessment of phylogeny, population structure, and genetic diversity

The sequences were next assembled and manually edited using SEQMAN™ (DNASTAR) to identify single nucleotide polymorphisms (SNPs) across all sequences. The representative cpDNA haplotype sequences of *H. littoralis* were registered in Genbank (accessions: MN729269–MN729290). To view intraspecific relationships among the cpDNA haplotypes, an un‐rooted network was constructed using the median‐joining approach implemented in NETWORK ver. 5.0.1.1 (Bandelt, Forster, & Röhl, 1999). Continuous indels were treated as single mutational events in the analysis. Phylogenetic relationships between the cpDNA haplotypes were reconstructed by maximum‐parsimony (MP) and maximum‐likelihood (ML) methods using PAUP ver. 4.0b10 (Swofford, 2003) and RAXML ver. 8.2.9 (Stamatakis, 2014), respectively. Analyses were conducted considering indels both as missing data and single mutational events. The program MODELTEST (Posada & Crandall, 1998) was used to select parameters and assumptions of ML analysis. The heuristic search parameters for both MP and ML were random addition of 1,000 replicates of sequence with tree‐bisection‐reconnection branch swapping and MULTREES and COLLAPSE options on. The relationships between haplotypes were supported by the estimated bootstrap values.

To evaluate the population structure, we used the spatial analysis of molecular variance (SAMOVA; Dupanloup, Schneider, & Excoffier, 2002) algorithm implemented in SPADS ver. 1.0 (Dellicour & Mardulyn, 2014). The SAMOVA algorithm uses a simulated annealing procedure to maximize the proportion of total genetic variance between groups of populations and defines groups which are geographically homogeneous and maximally differentiated from each other (Dupanloup et al., 2002). We considered models with putative numbers of populations (*K*) ranging from 1 to 10 and for each *K*, we used 1,000 simulations of annealing process for each of the 100 repeated runs. The samples were grouped under four populations (*K* = 4) for which largest genetic differentiation (*F*_CT_) was obtained (see Results and Table 1). Pairwise genetic distance between samples was estimated through two distance metrics, namely *D*_A_ (Nei, Tajima, & Tateno, 1983) and *F*_ST_ (Wright, 1951), using the program DNASP ver. 5.10 (Librado & Rozas, 2009). We used the genetic distance *D*_A_ to evaluate the genetic relationship between samples through a principal coordinate analysis (PCoA) using GENALEX ver. 6.5 (Peakall & Smouse, 2012). *F*_ST_ as a genetic distance matrix was used in BARRIER ver. 2.2 (Manni, Guerard, & Heyer, 2004) to implement the Monmonier's maximum difference algorithm for identifying the biogeographical boundaries exhibiting the largest genetic discontinuities between sample pairs. To detect isolation‐by‐distance (IBD) pattern among all populations, relationship between pairwise genetic distances [*F*_ST_/(1−*F*_ST_)] and geographic distances (log‐transformed values) was examined using Mantel tests (Mantel, 1967), as implemented in GENALEX, with 10,000 random permutations.

We calculated unbiased haplotype diversity corrected for sample size (Hd) and nucleotide diversity (*π*) following (Nei, 1987) for individual samples as well as for the populations (identified through SAMOVA) by using the program DNASP. Average gene diversity within populations (*H*_S_), total gene diversity (*H*_T_), coefficient of genetic variation over all populations (*G*_ST_), and coefficient of genetic variation influenced by both haplotype frequencies and genetic distances between haplotypes (*N*_ST_) were estimated for the populations using the program PERMUT (Pons & Petit, 1996). We used 1,000 permutation tests to assess the statistical significance of the difference between *G*_ST_ and *N*_ST_ in which significant *N*_ST_ > *G*_ST_ would indicate the presence of phylogeographic structure (Pons & Petit, 1996). Genetic differentiation between these populations was further examined through multiple hierarchical analyses of molecular variance (AMOVAs) with 1,000 permutations using ARLEQUIN (Excoffier, Smouse, & Quattro, 1992), considering – (a) all four populations without partitioning and (b) four populations individually.

### 2.3 Inferring demographic history

To investigate recent demographic expansion, we performed the Tajima's *D* test (Tajima, 1989) and Fu and Li's *F* test (Fu, 1997) in the program DNASP. For neutral markers, significant negative values of *D* and *F* would indicate recent population expansion (Hudson, 1990). In addition, we also conducted a mismatch distribution analysis (MDA) in ARLEQUIN to assess if the populations experienced past population expansions. Two metrics, namely the sum of squared deviations (SSD) between the observed and expected distributions and the raggedness index of Harpending [*H*_RAG_; Harpending, 1994] were used to validate the goodness‐of‐fit of the models.

**Figure 2:**
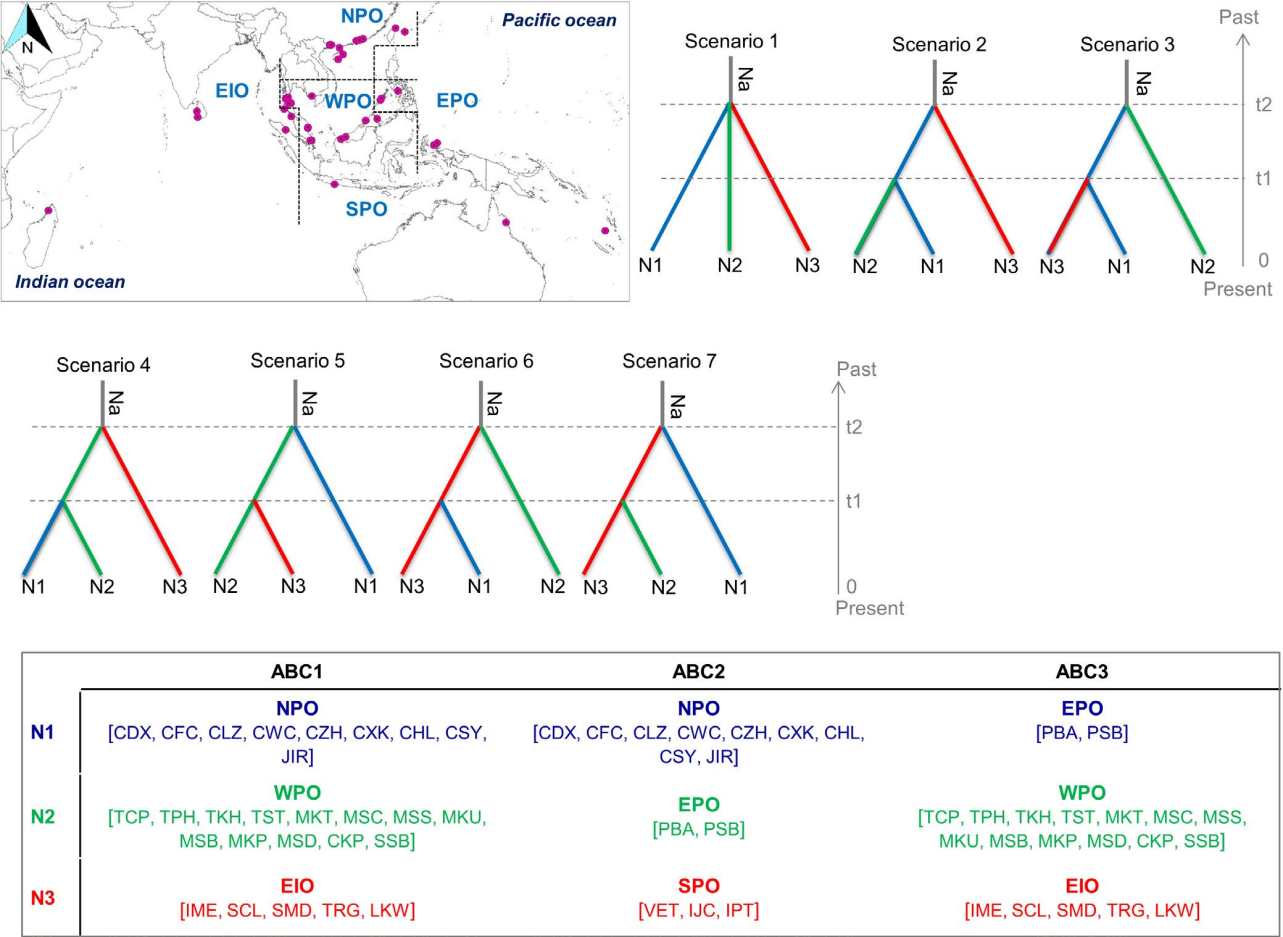
The seven scenarios tested for each of the two approximate Bayesian computation (ABC) models implemented in the software DIYABC ver.2.0. The population groups considered in each approach have been provided. In all scenarios, t# represents time scale measured in number of generations and N# represents effective population size of the corresponding populations during the relevant time period (e.g., 0–t1, t1–t2). Abbreviations of the sampling sites have been given in Appendix S1

The demographic history of divergence between populations was carried out on the SNPs using the approximate Bayesian computation (ABC) approach implemented in the software DIYABC ver.2.0 (Cornuet et al., 2014; Cornuet et al., 2008). To keep the scenarios simple and reduce the computational time, we conducted three ABC analyses (Figure 2). In the first analysis (hereafter, ABC1), we considered three populations consisting of samples having the major haplotypes (see Results)—population 1 consisting of samples from southern China and Japan of north Pacific Ocean (NPO), population 2 with samples located east of the Malay Peninsula in the west Pacific Ocean (WPO), and population 3 with the samples of the Strait of Malacca and Sri Lanka in the east Indian Ocean (EIO). The second ABC model (hereafter, ABC2) was conceptualized to determine the divergence time among populations of haplotype group 1 (see Results)—population 1 consisting of samples from NPO, population 2 with the Philippines samples of the east Pacific Ocean (EPO), and population 3 with the samples from the south Pacific Ocean (SPO). The final ABC model (hereafter, ABC3) was built for estimating the divergence time across SPO, EPO, and EIO.

In all model scenarios, t# represented time scale measured in the number of generations and N# represented the effective size of the corresponding population groups during the relevant time period (e.g., 0–t1, t1–t2). The standard Hudson's algorithm was applied as minimum allele frequency criterion to simulate the SNP data sets. We used default prior values for all parameters, except for maximum population size and the maximum values of time scale (100,000 instead of 10,000 default values) based on the findings of the preliminary test runs. Genic diversities, *F*_ST_ and Nei's distances were used as summary statistics for each of the three population groups. One million simulations were run for each scenario, and we chose the most‐likely scenario based on the comparative assessment of the posterior probabilities of the scenarios. We also checked the goodness‐of‐fit of the selected scenario through principal component analysis (PCA) using the “model checking” option in the software.

## 3 RESULTS

### 3.1 Phylogeny of the cpDNA haplotypes and their geographic distribution

**Figure 3:**
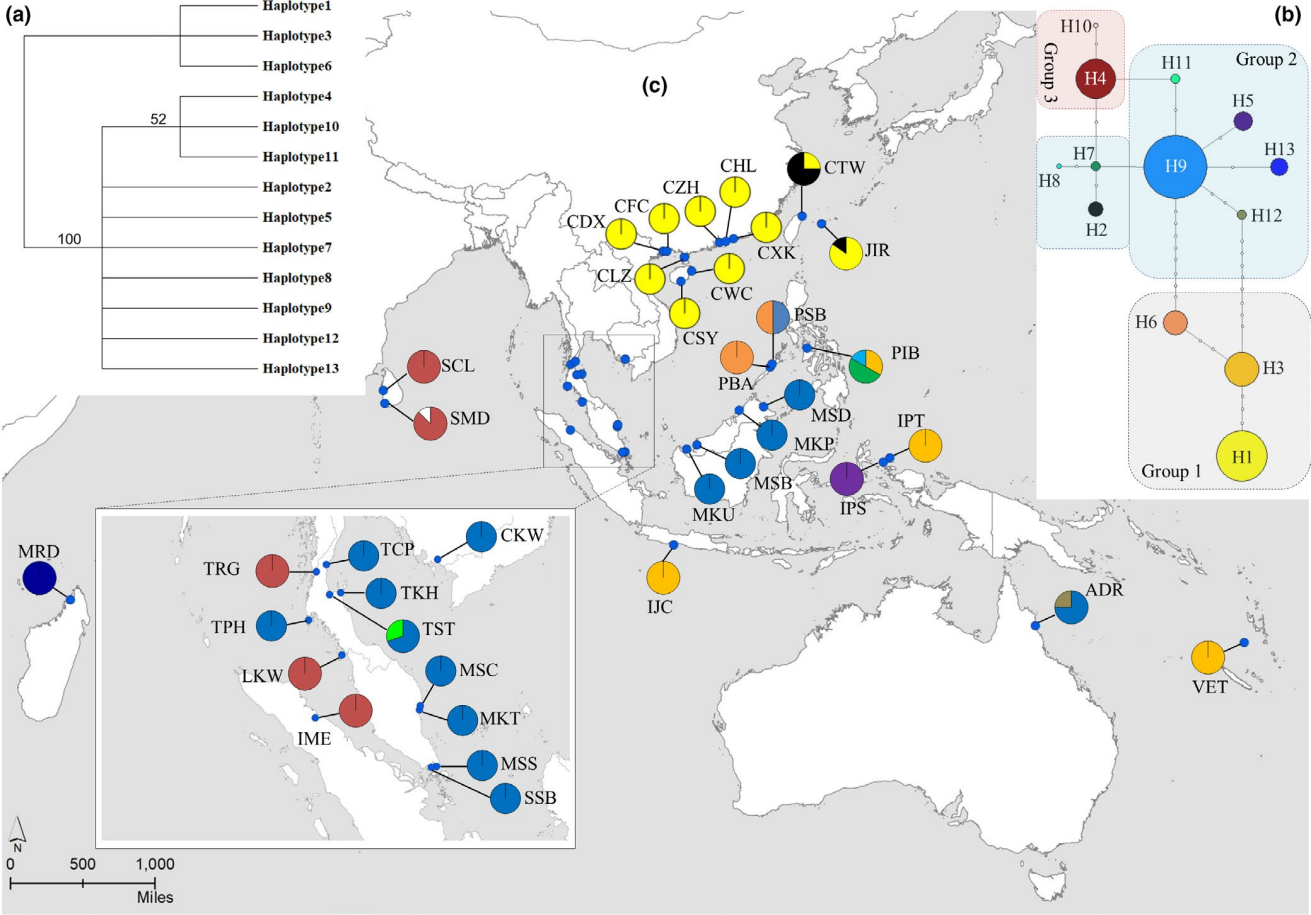
Relationship and distribution of 13 cpDNA haplotypes in *Heritiera littoralis*—(a) phylogenetic relationships of the haplotypes resolved through Maximum‐Parsimony method with numbers on branches showing the supporting ratio obtained from bootstrapping with 1,000 replicates; (b) median‐joining network for the haplotypes in which the size of the circle is proportional to the frequency of each sampled haplotype and the black dots on the branches indicate the number of steps separating adjacent haplotypes. Three hypothetical haplotype groups are indicated as group 1, 2, and 3; (c) geographical distribution of the haplotypes. Abbreviations of the sampling sites have been given in Appendix S1

Our study found a low proportion of polymorphic cpDNA regions (5 out of 24 loci; 20.83%) suggesting that chloroplast genome of *H. littoralis* is highly conserved. The alignment lengths of the five fragments were 760 bp for *atp*B*‐rbc*L, 975 bp for *acc*D*‐psa*I, 946 bp for *rpl*16, 894 bp for *trn*S‐G, and 876 bp for *trn*V‐M. In the total concatenated length of 4,451 bp, we found 19 polymorphic sites (6 singletons and 13 parsimony‐informative mutations) when including 5 long‐fragment indels, which corresponded to 13 haplotypes (H1–H13; Table 1; Appendix S3). The heuristic maximum‐parsimony (MP) search produced a single most parsimonious tree (length = 23 steps, RI = 0.852, CI = 0.892) (Figure 3a) when we recorded each long‐fragment indel as a single mutation event (site), which was topologically the same as the MP tree constructed on the sequence with indel as missing data (Appendix S4a) and the ML trees (Appendix S4b,d). We could identify three clades (haplotype 1/3/6, 4/10/11, and rest of the haplotypes). Relationship between clade 1/3/6 and all other haplotypes was resolved with high bootstrap support; however, low support was found for the relationship between clade 4/10/11 and rest of the haplotypes.

The NETWORK analysis grouped the 13 haplotypes into at least three haplotype groups (Figure 3b) in line with the MP tree of the phylogenetic relationship analysis. One haplotype group (group 1) comprised of H1 separated from H3 by three steps which were two steps distant from H6. The second haplotype group (group 2) was centered on H9 which was separated from 10 neighboring haplotypes by only 1–2 step distances. The two haplotypes (H4 and H10) were considered belonging to a separate group (group 3). The geographic distribution of these haplotypes showed that H1 was restricted to samples of southern China and Japan of north Pacific Ocean (NPO) whereas H4 was observed in the Strait of Malacca and east Indian Ocean (EIO) (Figure 3c). The samples of east of the Malay Peninsula (WPO) were found to have H9 haplotype which was separated by one step distance from H13 (east African Madagascar population) and by two sites from H12 (Northern Australia). High levels of haplotype diversity were observed in the samples of the Palawan and Philippines of the eastern Pacific (EPO) with 5 haplotypes (H3, H6–H9) whereas the haplotypes H3 and H5 were found in the southern Pacific (SPO).

### 3.2 Population structure and genetic diversity

The SAMOVA revealed the highest *F*_CT_ value (0.894) when the 37 samples were divided into four populations (Table 1), being nearly consistent with the major haplotype distribution. Geographically, the samples from southern China and Japan formed one cluster (hereafter, POP I) whereas samples of east of the Malay Peninsula formed another cluster with the samples from Taiwan, east Africa, and Australia (hereafter, POP II). The samples of the southern Pacific (except PIB and IPS) formed a separate cluster with the Palawan and Philippines samples (POP III) whereas the samples from east Indian Ocean and Strait of Malacca were found to belong to a separate cluster (POP IV).

**Figure 4:**
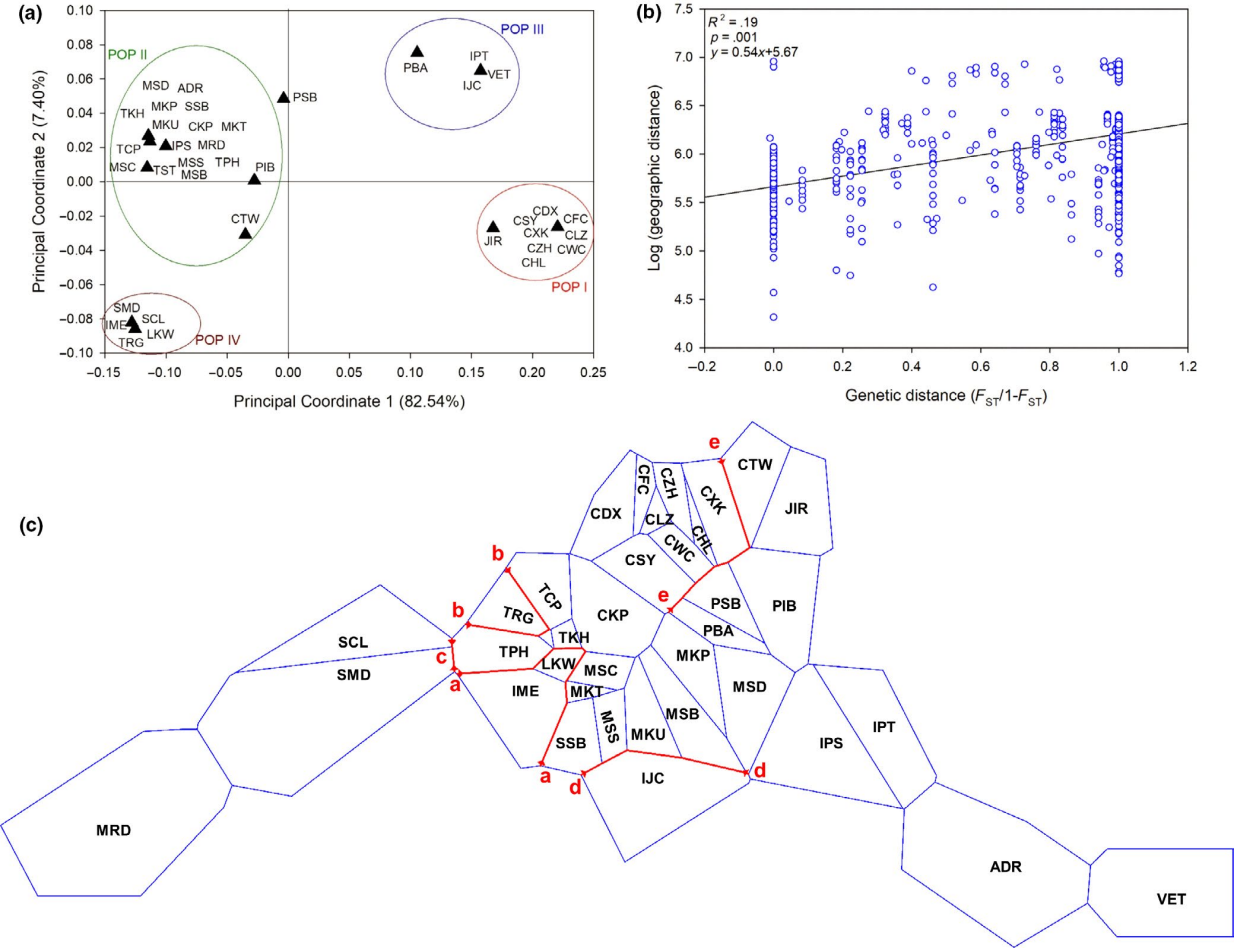
Relationship between geography and genetic differentiation across sampling locations of *Heritiera littoralis* in the IWP—(a) Principal coordinate analysis (PCoA) grouping 37 sampling sites into four groups, generally consistent with the population clusters identified by SAMOVA, (b) Scatterplot of Mantel test showing relationship between pairwise genetic and geographic distances, and c) Potential gene flow barriers, represented with red lines, between the sampling sites around each of which the blue polygons depict the Voronoï tessellation. Abbreviations of the sampling sites have been given in Appendix S1

In the PCoA analysis, the first two axes could explain maximum variation (89.95%) along which four separate and distinct population clusters were observed (Figure 4a), being consistent with the SAMOVA and the haplotype aggregation analysis. The samples from southern China and Japan (POP I, primarily consisting of H1) were found to be distant from the population cluster formed by the samples of the east of the Malay Peninsula, Taiwan, east Africa, and Australia (POP II having group 2 haplotypes), and samples from the east Indian Ocean and Strait of Malacca (POP IV with group 3 haplotypes). Along the X‐axis which could explain maximum variation (82.54%), POP I was relatively close to POP III (having H3 and H6 haplotypes from haplotype group 1). Although POP II and POP IV were found to be close along the X‐axis in the PCoA analysis, the Monmonier's algorithm implemented in the BARRIER analysis identified potential geographical barriers between these populations (barriers a, b, and c) and between POP I and POP II (barrier e), suggesting genetic isolation between them (Figure 4c). Overall population differentiation (*F*_ST_) was found to be weakly related to geographic distance (*r* ^2^ = 0.19, *p* < .05) as revealed from the Mantel test (Figure 4b). We found no significant correlation between genetic and geographic distances for the four populations.

Haplotype diversity (Hd) and nucleotide diversity (*π*) were found to be 0.786 and 0.343 respectively across the distribution range of *H. littoralis*. High genetic diversity was observed in POP III (Hd = 0.565; *π* = 0.135) and POP II (Hd = 0.435; *π* = 0.057) (Table 1). Total genetic diversity (*H*_T_ = 0.79 ± 0.04) was much higher than the average intrapopulation diversity (*H*_S_ = 0.098 ± 0.03) (Table 2), suggesting that the majority of cpDNA diversity is distributed among populations. Similar to Hd and *π*, *H*_T_ and *H*_S_ were found to be much higher in POP III (*H*_T_ = 0.65, *H*_S_ = 0.11) and POP II (*H*_T_ = 0.524, *H*_S_ = 0.168). Overall population differentiation was high (*G*_ST_ = 0.898, *N*_ST_ = 0.911) across its distribution range (Table 2), although the difference between *N*_ST_ and *G*_ST_ was not significant (*p* = .29). Hierarchical AMOVA analysis revealed that 89.46% variation can be attributed to the differentiation among these four population groups (Table 3), suggesting the geographical divergence of *H. littoralis* in the studied region. The pairwise *F*_CT_ values ranged from 0.66 to 0.97, showing high levels of genetic differentiation between these populations. When we considered the individual populations, maximum genetic variation was found to be within samples (POP I = 94.9%, POP II = 53.04%; POP IV = 95.37%) except POP III in which 67.85% variation was attributed to the differentiation among samples (Table 3).

**Table 2:** Results of genetic diversity analysis conducted to estimate average genetic diversity within populations (*H*_S_), total genetic diversity (*H*_T_), interpopulation differentiation (*G*_ST_), and the number of substitution types (*N*_ST_) for the four populations identified in the spatial analysis of molecular variance (SAMOVA)

Region	*H*_S_	*H*_T_	*G*_ST_	*N*_ST_
Overall	0.098 ± 0.03	0.788 ± 0.04	0.876	0.950
POP I	0.031 ± 0.03	0.034 ± 0.03	0.083	0.083
POP II	0.168 ± 0.07	0.524 ± 0.13	0.680	0.758
POP III	0.108 ± 0.11	0.650 ± 0.17	0.834	0.692
POP IV	0.050 ± 0.01	0.050 ± 0.01	1.330	0.000

**Table 3:** Results of the analysis of molecular variance (AMOVA) performed considering the four populations identified in the spatial analysis of molecular variance (SAMOVA)

Source	*df*	Sum of squares	Variance components	Percentage of variation
All populations				
Among populations	3	1,016.12	3.872	87.01
Among samples within populations	33	103.69	0.286	6.41
Within samples	338	98.86	0.293	6.57
Total	374	1,218.67	4.450	
POP I				
Among samples	8	3.93	0.017	5.06
Within samples	78	25.39	0.325	94.94
Total	86	29.31	0.343	
POP II				
Among samples	17	46.56	0.262	46.96
Within samples	151	44.60	0.295	53.04
Total	168	91.16	0.557	
POP III				
Among samples	4	53.09	1.001	67.85
Within samples	59	28.00	0.475	32.15
Total	63	81.09	1.476	
POP IV				
Among samples	4	0.11	0.009	4.63
Within samples	50	0.88	0.018	95.37
Total	54	0.98	0.019	

### 3.3 Inference of demographic history

The mismatch distribution analysis revealed non‐significant SSD and *H*_RAG_ values for POP II and POP IV (Table 4), and the observed curves were not of typical unimodal distribution (Appendix S5). Although Tajima's *D* was negative for both populations, the value was non‐significant for POP IV (Table 1). The Fu's *F*s statistic which is more sensitive to population demographic expansion also showed low negative values for both the populations. The significance tests for SSD and *H*_RAG_ were not uniform for POP I (SSD = 0.003, *p* = .07; *H*_RAG_ = 0.915, *p* = .84) and POP III (SSD = 0.475, *p* < .05; *H*_RAG_ = 0.501, *p* = .96) (Table 4). The neutrality tests for POP I showed significantly negative Tajima's *D* but positive *F*s, and positive Tajima's *D* and *F*s for POP III. Overall, these findings could not provide strong evidence of demographic expansion of *H. littoralis* in this region.

**Table 4:** Results of mismatch distributions analysis and neutrality test (Tajima's *D*, Fu's *F*s tests) for the four populations identified in the spatial analysis of molecular variance (SAMOVA)

Region	Mismatch distribution		Neutrality tests	
	SSD (*p*‐value)	*H*_RAG_ (*p*‐value)	Tajima's *D* (*p*‐value)	Fu's *F*s
All populations	0.0479 (.01)	0.0993 (.11)	3.074 (<.01)	8.650
POP I	0.0031 (.07)	0.9153 (.84)	−2.174 (<.01)	3.098
POP II	0.0045 (.17)	0.1256 (.63)	−1.822 (<.05)	−2.802
POP III	0.4753 (<.01)	0.5014 (.96)	0.595 (>.10)	6.875
POP IV	0.0010 (.17)	0.8612 (.86)	−1.093 (>.10)	−1.716

Abbreviations: *H*_RAG_, Harpending's raggedness index; SSD, Sum of squared deviations.

Comparing the scenarios for three ABC models, the highest values of posterior probability were obtained for scenario 7 in ABC1 (0.426; 95% CI: 0.41–0.49), scenario 3 in ABC2 (0.563; 95% CI: 0.45–0.68) and in ABC3 (0.557; 95% CI: 0.48–0.63), and did not overlap with the 95% CI of other scenarios (Appendix S6). Absence of significant differences between the observed and simulated data in most of the 36 summary metrics (Appendix S7) and the position of the observed data in close proximity of the simulated data cluster in the PCA (Appendix S8) showed that the selected scenarios were good fit for the observed data. In ABC1, the median values of the effective population size of N1 (NPO), N2 (WPO), N3 (EIO), and NA (putative ancestral population) were 90,000, 2,550, 34,800, and 92,700, respectively (Table 5). The divergence times when N2 split from the N3 (t1) and from N1 (t2) were estimated to be 41,000 (95% CI = 8,800–85,400) and 68,000 (95% CI = 18,700–98,100) generations ago, respectively. With an approximate estimate of the generation time of *H. littoralis* around 20 years, the divergence times of t1 and t2 were converted into absolute time of 0.82 and 1.36 MYA, respectively. In ABC2, the median values of the effective population size of N1 (NPO), N2 (EPO), N3 (SPO), and NA (putative ancestral population) were 74,000, 10,800, 38,200, and 76,300 respectively (Table 5). The divergence times when N2 split from the N3 (t1) and from N1 (t2) were estimated to be 26,100 (95% CI = 7,000–72,900) and 74,000 (95% CI = 28,400–98,700) generations ago, respectively. With an approximate estimate of the generation time of *H. littoralis* around 20 years, the divergence times of t1 and t2 were converted into absolute time of 0.52 and 1.48 MYA, respectively. In ABC3, the median values of the effective population size of N1 (EPO), N2 (WPO), N3 (EIO), and NA (putative ancestral population) were 79,000, 5,250, 23,300, and 88,600, respectively (Table 5). The divergence times when N2 split from the N3 (t1) and from N1 (t2) were estimated to be 16,000 (95% CI = 3,470–57,100) and 63,100 (95% CI = 15,700–97,700) generations ago, respectively. With an approximate estimate of the generation time of *H. littoralis* around 20 years, the divergence times of t1 and t2 were converted into absolute time of 0.32 and 1.26 MYA, respectively.

**Table 5:** Estimated divergence parameters for three ABC models conceptualized for this study

Model	Parameters	Mean	Median	Mode	95% Confidence interval (lower–upper)
ABC1	N1	87,000	90,000	92,500	55,700–98,200
	N2	4,760	2,550	1,570	231–25,900
	N3	36,300	34,800	24,000	4,740–75,500
	t1	42,700	41,000	38,500	8,880–85,400
	t2	65,000	68,000	75,900	18,700–98,100
	NA	87,900	92,700	99,500	48,600–99,700
ABC2	N1	71,900	74,000	79,100	35,000–97,200
	N2	16,100	10,800	5,040	1,370–64,000
	N3	42,400	38,200	31,100	8,640–94,400
	t1	29,800	26,100	20,100	7,000–72,900
	t2	71,600	74,000	90,100	28,400–98,700
	NA	70,500	76,300	94,600	16,900–98,900
ABC3	N1	77,100	79,000	82,200	43,300–98,200
	N2	7,110	5,250	2,970	644–25,200
	N3	27,000	23,300	17,400	3,700–69,500
	t1	19,700	16,000	9,770	3,470–57,100
	t2	61,100	63,100	66,000	15,700–97,700
	NA	83,700	88,600	98,300	38,200–99,600

Abbreviations: N#, Effective population size; t#, time scale measured in the number of generations.

## 4 DISCUSSION

Using chloroplast DNA sequences, our study estimated genetic diversity and population structure of *H. littoralis*, and identified how glacial vicariance, contemporary ocean currents, and long‐distance dispersal events might have influenced the current distribution of the species in the Indo‐West Pacific region.

### 4.1 Low genetic diversity and prominent genetic structure of *H. littoralis* in the IWP

Although a relatively high level of genetic diversity at the species level (*H*_T_ = 0.79) was observed for *H. littoralis* in accordance with previous studies (Jian et al., 2004, 2010), we found low genetic diversity at the population level across its entire distribution range. No polymorphism was detected within the populations from southern China, Malaysia, and Indonesia. Although local diversity may occasionally be inflated by genetic admixture from other differentiated populations, we found little evidence of genetic exchange between geographically and genetically distinct populations. Rather, we identified strong genetic structure associated with geography in *H. littoralis* from the genetic clustering analyses (SAMOVA, PCoA) which showed, more or less unanimously, that the distributional range of the species can be divided into at least four population clusters. The haplotype composition was different in these population clusters and phylogenetic differentiation between the frequently observed haplotypes was revealed by the median‐joining NETWORK analysis. Furthermore, high pairwise *F*_CT_ values (0.68–0.97) along with maximum proportion of variation due to differentiation among population clusters were found through AMOVA analysis. Although we observed high levels of cytoplasmic structure (*G*_ST_/*N*_ST_ = 0.898/0.911), the non‐significance (*p* > .05) of this phylogeographic structure suggested that historical disruptions of gene flow might have influenced the lineage composition of contemporary populations of *H. littoralis* in the IWP region. Overall, these findings indicated impediment of genetic exchange between populations of *H. littoralis* in the IWP, as observed for many mangroves (e.g., Binks et al., 2019; Guo, Ng, et al., 2018) and other species having water dispersed propagules (Yamamoto et al., 2019).

This population structure could be generated by three major barriers to gene flow, namely the geographic distance, land masses, and ocean currents. We found a weak relationship between geographic and genetic distance in *H. littoralis*, being consistent with the isolation‐by‐distance (IBD) pattern found in other mangroves like *Rhizophora mangle* (Cisneros‐de la Cruz et al., 2018). This finding suggested that population structure of this species cannot be fully explained by a single stepping‐stone model (Cerón‐Souza et al., 2015), and given that IBD is often biased by hierarchical population structure (Ngeve, Van Der Stocken, Menemenlis, Koedam, & Triest, 2016), the spatial distribution of genetic variance was more likely caused by effective physical barriers to gene flow like glacial vicariance and contemporary oceanic circulation pattern.

### 4.2 Glacial vicariance and genetic differentiation

From the haplotype composition, we found distinct lineages dominated different oceanic regions—H1 in the NPO, H3 in the SPO, H6 in the EPO, H9 in the WPO, and H4 in the EIO. The phylogenetic relationships from the ML and MP trees and the NETWORK analysis of these haplotypes revealed that H1 and H3 shared common ancestry along with H6 which was further related to H9 and H4 haplotypes. The inferred divergence times through ABC analyses suggested that H6 diverged from H1 and H3 haplotypes in around 1.48 MYA (95% CI = 0.57–1.97 MYA) and from H9 and H4 haplotypes in around 1.26 MYA (95% CI = 0.31–1.95 MYA). Furthermore, we observed close relationship between the haplotypes of NPO (H1) and SPO (H3) which diverged in around 0.52 MYA (95% CI, 0.14–1.46 MYA). This weak genetic differentiation between Indo‐Malesia and Australasia is in contrast with other studies which identified plate tectonic movements of the Indo‐Australian Archipelago (IAA) as one of the factors causing high genetic differentiation between these regions for many mangrove species [e.g., Urashi, Teshima, Minobe, Koizumi, & Inomata, 2013). The temporal scale of these divergence events indicated that these haplotypes were related in ancient times until the Pleistocene epoch (1.5 MYA) when the repeated emergence and submergence of land masses due to glacial sea level changes might cause divergence of these haplotypes. One of the major land masses which were exposed during this time period was the Malay Peninsula (Hall, 2009) which historically impeded gene flow between the Pacific and Indian Oceans (Lohman et al., 2011; Voris, 2000). Our findings also indicated that emergence of this land mass might cause the divergence between H9 of WPO and H4 of EIO in around 0.82 MYA (95% CI = 0.18–1.71 MYA). The Monmonier's algorithm identified the Malay Peninsula as one of the geographic barriers in this region, suggesting its ongoing role in impediment of genetic exchange between the Pacific and Indian Oceans. These findings, therefore, add evidence to the growing literatures which identified Pleistocene glacial vicariance as one of the important phylogeographic forces shaping the current distribution pattern of many mangroves (e.g., Duke et al., 2002; Urashi et al., 2013; Yang et al., 2017) and other marine species (e.g., Gaither et al., 2011) in the IWP.

Overall, these findings indicated that *H. littoralis* might have been established in the IWP recently by the rapid expansion of small number of founder populations from the EPO region. Besides having the H6 haplotype, the sampling locations of this region were found to have high haplotype and nucleotide diversities, thereby suggesting possible presence of refugia populations of *H. littoralis* in these regions. The glacial phases of Pleistocene caused loss of suitable habitats and environments for the mangroves in the IWP, and confined them near the equator (Hodel, Cortez, Soltis, & Soltis, 2016). These populations which persisted throughout glacial maxima in refugia are often characterized with higher genetic diversity and/or spatially patterned genetic differentiation (Provan & Bennett, 2008), as observed for the EPO population. Recent range expansion of *H. littoralis* in the IWP was further supported by the mismatch analysis which showed unimodal profiles and non‐significant SSD and *H*_RAG_ values for most of the populations in this region. This might also explain the observed low genetic diversity of *H. littoralis* in this region which has been often attributed to repeated extinction‐recolonization events due to continuous sea level changes during the Pleistocene epoch. Similar paucity of intraspecific genetic diversity due to steep bottlenecks during glaciations and founder effects during recolonization was also found for other mangroves (e.g., *Avicennia marina*; Arnaud‐Haond et al., 2006), *Bruguiera gymnorrhiza*; Urashi et al., 2013).

### 4.3 Oceanic circulation pattern and transoceanic dispersals

Although these genetic barriers caused genetic differentiation among geographic regions, our study found evidence of long‐distance dispersal of *H. littoralis* propagules across these barriers. First, one of the populations located at the west coast of the Malay Peninsula (TPH) was found to be genetically similar to the east of the peninsula. In this region, corridors like the Strait of Malacca opened during the short interglacial period with the sea levels rising (Voris, 2000), where part of the sea surface current enters from the SCS and flows northward along the west coast of the Malay Peninsula and leaves south of the Andaman Islands (Mansor et al., 2018). This oceanic circulation pattern may provide opportunities for infrequent LDD detouring around the Strait, as has been observed for other mangroves (Yang et al., 2017), and provide a plausible explanation of genetic exchange across the peninsula. Secondly, the central haplotype H9 of the WPO was also found in northeast Australia (ADR) of the SPO. Although the Indo‐Australian Archipelago plays an important role in the genetic differentiation between Indo‐Malesia and Australasia, it was seldom considered the strongest genetic break range wide, except for in *Rhizophora stylosa* (Wee et al., 2015). Indeed, considerable gene flow between these two regions has been observed being mediated by the Indonesian throughflow (ITF) which moves from the Celebes Sea through the Makassar Strait to the Timor Sea (Figure 1). Thus the ITF might have acted as a major corridor for southward sea‐drifted gene flow during the Pleistocene glaciations (Li et al., 2016). Thirdly, in the southern Pacific, the South Equatorial Counter Current flowing through north of New Guinea to Halmahera may facilitate northward dispersal of propagules which could explain the shared haplotype (H3) between Vanuatu (VET) of SPO and east Indonesia (IPT) of EPO. We found the same haplotype (H3) in southern Java (IJC) of SPO which might have been introduced from Philippines (PIB) or east Indonesia (IPT) of EPO through the ITF exiting through the Lombok Strait between the islands of Bali and Lombok (Figure 1).

In addition to intraoceanic dispersal, LDD ability of *H. littoralis* might help the species to expand its range through transoceanic dispersal to the eastern Africa (EA) in the Indian Ocean. Our findings indicated that the minor seasonal flow via the South China Sea (SCS) exiting either to the Timor Sea or the Lombok Strait might carry the propagules of the WPO through the South Equatorial Current across the Indian Ocean to the EA (Figure 1), as evident from the close relationship between the haplotypes found in these regions (H9 and H13). Recent studies have suggested that the Indian Ocean has no longer been an effective dispersal barrier for mangroves, especially for those having floating period more than six months (Van Der Stocken, Carroll, et al., 2019). Long‐distance dispersal of propagules through Indian South Equatorial Current and close relationship between east African and Australian populations has been established for mangrove species like *R. mucronata* (Lo, Duke, & Sun, 2014). Given that the floating period of *H. littoralis* seeds (150 days) is one of the longest floating periods of the mangroves (Van Der Stocken, Wee, et al., 2019), it is likely that direct (i.e., not stepping‐stone) connectivity between coastal regions in the WPO and EA could have been established through LDD across the Indian Ocean.

While the ocean circulation pattern facilitated genetic exchange across the land barrier, it was found to act as a barrier impeding genetic exchange within the SCS, as evident from the presence of two different lineages within the same oceanic region. In this region, *H. littoralis* fruits in summer (from June to September) when the surface current of the tropical Indian Ocean flows northward into the South China Sea and then toward the Pacific Ocean through the Bashi Strait (Fang, Wang, Fang, & Fang, 2012). The seeds of the Malay Peninsula can reach the coasts of Philippines and Taiwan, but the dispersal is limited to southern China. On the other hand, the southern China populations can be dispersed to Japan through a north‐eastward current which originates from the southeast of the Hainan Island and extends toward the East China Sea through the Taiwan Strait. This circulation pattern of the SCS Throughflow might therefore hinder any genetic exchange and cause genetic differentiation in this region. Presence of similar inconspicuous genetic barrier was also observed in the phylogeographic pattern of other mangroves (e.g., *R. stylosa*; Wee et al., 2015 and *R. apiculata*; Guo et al., 2016).

### 4.4 Conservation implications

Although *H. littoralis* is considered as a species of least concern by the IUCN, human activities like overexploitation for wood, ornamental and medicinal values (Tomlinson, 2016), and habitat degradation from urbanization and pollution are threatening the survival of the species across its distribution range. In fact, most of the samples for this study were collected from protected areas and natural reserves since its natural habitats along the coast have been destroyed by anthropogenic activities. With poor reproductive capacity in terms of low seed production, rate of germination and transformation of juvenile to adult (Jian et al., 2010), the species may face a serious threat of extinction in near future. In this context, the intraspecific population genetic structure revealed by this study can be translated to conservation implications by preserving the evolutionary potential of the species and maintaining as much genetic diversity as possible. Our findings showed that haplotype diversity of the species is centered in few geographic areas of its distribution range and the relatively strong genetic differentiation among the populations emphasizes need for comprehensive conservation program for all populations across its distribution range. Furthermore, our study revealed low genetic polymorphism at the intraspecific level across its distribution range which can result in a reduction in the capacity to cope with global climate changes. Therefore, a long‐term in situ conservation program is necessary to maintain areas with high genetic diversity and to restore disturbed habitats to prevent further extinction. Trading of live plants across its distribution range may also help to reduce genetic erosion; however, caution will be required while introducing foreign germplasms as it may disrupt local adaptation, spread deleterious alleles, or cause outbreeding depression (Su, Wang, & Deng, 2010).

## 5 CONCLUSION

Our study revealed relatively low genetic diversity and prominent population structure of *H. littoralis* in the IWP, primarily caused by geographic barriers to gene flow which were generated by sea level changes over the past several hundred thousand years. However, variable number of sample sizes, primarily due to fragmented distribution of the species in the IWP, might have affected the estimated diversity parameters (Leberg, 2002). The ABC inferences of divergence times between regions might also suffer from uncertainties like generation time of the species, overlapping of generations and 95% CI of the inferred parameters, and the temporal estimates can also be biased as they ignore gene flow after divergence (Tsuda, Nakao, Ide, & Tsumura, 2015). Furthermore, although the LDD ability of the *H. littoralis* propagules helped the species to occasionally cross these genetic barriers, it is difficult to distinguish these events from incomplete lineage sorting, and therefore, the observed inter‐regional gene flow should be interpreted carefully (Ng et al., 2015). Nevertheless, fairly equal number of individuals sampled across most of the sites, very low polymorphism and considering the variation of sample sizes in the estimation of diversity parameters might minimize the biasness generated from the sampling size variation. Given the high genetic differentiation and limited evidence of haplotype sharing between populations of east and west of the Malay Peninsula as well as between Indo‐Malesia and Australasia, the bias of temporal estimates might be limited. No consideration of gene flow after divergence may also underestimate divergence times between populations; however, with the population split occurring before the Last Glacial Maximum (LGM, ca. 20,000 years BP) would not have changed the main discussion here.

## CONFLICT OF INTEREST

None declared.

## AUTHOR CONTRIBUTION

**Achyut Kumar Banerjee:** Data curation (equal); Formal analysis (equal); Investigation (lead); Methodology (equal); Software (equal); Validation (equal); Visualization (lead); Writing‐original draft (lead); Writing‐review & editing (lead). **Wuxia Guo:** Conceptualization (equal); Formal analysis (supporting); Investigation (equal); Methodology (equal); Project administration (equal); Resources (equal); Validation (equal); Writing‐original draft (supporting). **Sitan Qiao:** Data curation (equal); Formal analysis (equal); Investigation (equal); Methodology (equal); Software (equal). **Weixi Li:** Data curation (equal); Investigation (equal); Methodology (equal); Resources (equal); Software (equal); Validation (equal). **Fen Xing:** Data curation (equal); Formal analysis (equal); Investigation (equal); Methodology (equal); Resources (equal). **Yuting Lin:** Data curation (equal); Formal analysis (equal); Investigation (equal); Methodology (equal); Software (equal); Validation (equal). **Zhuangwei Hou:** Data curation (equal); Formal analysis (equal); Investigation (equal); Methodology (equal); Software (equal); Validation (equal); Visualization (equal). **Sen Li:** Conceptualization (equal); Formal analysis (equal); Investigation (equal); Methodology (equal); Validation (equal); Visualization (equal). **Ying Liu:** Conceptualization (equal); Project administration (equal); Resources (equal); Supervision (equal); Visualization (equal); Writing‐review & editing (supporting). **Yelin Huang:** Conceptualization (lead); Funding acquisition (equal); Investigation (equal); Project administration (lead); Resources (equal); Supervision (lead); Validation (equal); Visualization (equal); Writing‐original draft (equal); Writing‐review & editing (equal).

## Supporting information

Appendix S1

Appendix S2

Appendix S3

Appendix S4

Appendix S5

Appendix S6

Appendix S7

Appendix S8

## Data Availability

All relevant data are available as manuscript Appendices S1–S8, and sequences are deposited on the GenBank repository under the accession numbers MN729269–MN729290.
