# Supplementary material for: Land masses and oceanic currents drive population structure of Heritiera littoralis, a widespread mangrove in the Indo‐West Pacific
Source: Ecol Evol. 2020 Jun 3;10(14):7349–63. doi: 10.1002/ece3.6460 (PMC7391321; doi:10.1002/ece3.6460)
Supplement: Supplementary file 1 — Appendix S1 [file ECE3-10-7349-s001.docx]

| Country | Sampling location | ID | Latitude | Longitude |
| --- | --- | --- | --- | --- |
| Australia | Daintree river | ADR | -16.000 | 145.300 |
| Cambodia | Kamport | CKP | 10.717 | 104.183 |
| China | Dongxing, Guangxi | CDX | 21.533 | 107.967 |
|  | Fangchenggang, Guangxi | CFC | 21.517 | 108.333 |
|  | Leizhou, Guangdong | CLZ | 20.900 | 110.083 |
|  | Wenchang, Hainan | CWC | 19.533 | 110.817 |
|  | Zhuhai, Guangdong | CZH | 22.417 | 113.600 |
|  | Xiangkeng, Guangdong | CXK | 22.800 | 115.017 |
|  | Lizhiwo, Hongkong | CHL | 22.517 | 114.250 |
|  | Sanya, Hainan | CSY | 18.483 | 109.750 |
|  | Aodi, Xinbei,Taiwan | CTW | 25.050 | 121.917 |
| Indonesia | Sawinggrai, Papua | IPS | 0.400 | 130.033 |
|  | Cilacap, Central Java | IJC | -7.900 | 109.017 |
|  | TangangPutus, Papua | IPT | 0.817 | 130.700 |
|  | Medan | IME | 3.583 | 98.667 |
| Japan | Iriomote-jima | JIR | 24.283 | 123.867 |
| Madagascar | Ambanja | MRD | -13.430 | 48.500 |
| Malaysia | Sg.Balok, Kuantan | MKT | 3.950 | 103.367 |
|  | Sungai Cherating | MSC | 4.133 | 103.400 |
|  | Sungai Santi | MSS | 1.400 | 104.133 |
|  | Kuching | MKU | 1.667 | 110.333 |
|  | Sibu | MSB | 2.100 | 111.350 |
|  | Kuala Penyu | MKP | 5.567 | 115.600 |
|  | Sandakan | MSD | 5.917 | 118.033 |
|  | Lankawei | LKW | 6.417 | 99.867 |
| Philippines | Ibajay, Aklan | PIB | 11.817 | 122.400 |
|  | Bacongan | PBA | 9.883 | 118.717 |
|  | Sabang, Palawan | PSB | 10.217 | 118.900 |
| Singapore | Sungei Buloh Wetland Reserve | SSB | 1.367 | 103.900 |
| Sri Lanka | Chilaw | SCL | 7.550 | 79.900 |
|  | Madu Ganga River | SMD | 6.283 | 80.050 |
| Thailand | Chumphon | TCP | 10.483 | 99.167 |
|  | Phuket | TPH | 7.983 | 98.367 |
|  | Khanom | TKH | 9.217 | 99.817 |
|  | Surat Thani | TST | 9.133 | 99.317 |
|  | Ranong | TRG | 10.167 | 98.717 |
| Vanuatu | Efate island | VET | -17.700 | 166.267 |

**Appendix S1**: Details of 37 sampling locations of *Heritiera littoralis* in the IWP
