# Supplementary material for: Land masses and oceanic currents drive population structure of Heritiera littoralis, a widespread mangrove in the Indo‐West Pacific
Source: Ecol Evol. 2020 Jun 3;10(14):7349–63. doi: 10.1002/ece3.6460 (PMC7391321; doi:10.1002/ece3.6460)
Supplement: Supplementary file 2 — Appendix S2 [file ECE3-10-7349-s002.pdf]

Appendix S2: Sequences of the primers used for amplification of five non-coding cpDNA regions in *Heritiera littoralis*

Chloroplast Region	Primers used in this study	Sequences	References
*acc*D-*psa*I spacer	*accD*-769F	GGAAGTTTGAGCTTTATGCAAATGG	Barkman and Simpson (2002)
	psaI-75R	AGAAGCCATTGCAATTGCCGGAAA	
*trn*S-*trn*G spacer	*trn*S(GCU)	GCCGCTTTAGTCCACTCAGC	Hamilton (1999)
	trnG(UCC)	GAACGAATCACACTTTTACCAC	
*trn*V-*trn*M spacer	*trn*V	GCTATACGGGCTCGAACC	Cheng et al. (2005)
	*trn*M	TACCTACTATTGGATTTGAACC	
*rpl*16 spacer	*rpl*16-RF-int	GTAAGGKCTATGAAGCATCTMATAAAGAGC	Cronn et al. (2002)
	*rpl*16-R1516	CCCTTCATTCTTCCTCTATGTTG	
*atp*B-*rbc*L spacer	*atp*B	GTGGAAACCCCGGGACGAGAAGTAGT	Hodges and Arnold (1994)
	*rbc*L	ACTTGCTTTAGTTTCTGTTTGTGGTGA	

References:

Barkman TJ, Simpson BB. 2002. Hybrid origin and parentage of *Dendrochilum acuiferum* (Orchidaceae) inferred in a phylogenetic context using nuclear and plastid DNA sequence data. *Systematic botany,* **27**: 209-220.

Cheng Y-P, Hwang S-Y, Lin T-P. 2005. Potential refugia in Taiwan revealed by the phylogeographical study of *Castanopsis carlesii* Hayata (Fagaceae). *Molecular Ecology,* **14**: 2075-2085.

Cronn RC, Small RL, Haselkorn T, Wendel JF. 2002. Rapid diversification of the cotton genus (*Gossypium*: Malvaceae) revealed by analysis of sixteen nuclear and chloroplast genes. *American Journal of Botany,* **89**: 707-725.

Hamilton MB. 1999. Four primer pairs for the amplification of chloroplast intergenic regions with intraspecific variation. *Molecular ecology,* **8**: 521-523.

Hodges SA, Arnold ML. 1994. Columbines: a geographically widespread species flock. *Proceedings of the National Academy of Sciences,* **91**: 5129-5132.
