# Supplementary material for: Land masses and oceanic currents drive population structure of Heritiera littoralis, a widespread mangrove in the Indo‐West Pacific
Source: Ecol Evol. 2020 Jun 3;10(14):7349–63. doi: 10.1002/ece3.6460 (PMC7391321; doi:10.1002/ece3.6460)
Supplement: Supplementary file 3 — Appendix S3 [file ECE3-10-7349-s003.pdf]

Appendix S3: Variable sites of the aligned sequences of five chloroplast DNA fragments in 13 haplotypes of *H. littoralis*. Insertion-deletion sites are indicated by "-"

Haplotypes	***acc*D-*psa*I spacer**				***trn*V-*trn*M spacer**			***trn*S-*trn*G spacer**					***rpl*16 spacer**						***atp*B-*rbc*L**
	120-241	482	699-704	714-718	136	338	701	76-86	210-216	230-235	794	850	351	433	446	496	525	595	232
Hap_1	△*	C	-----	-----	T	T	G	-----------	-----------------	------	T	C	G	C	A	T	T	T	G
Hap_2	-	A	-----	ATAAT	G	G	T	-----------	TATACAACTTTTCTATA	ACTTTT	C	T	G	T	T	A	A	T	T
Hap_3	△	C	-----	ATAAT	G	T	G	-----------	-----------------	------	T	C	G	C	A	A	T	T	G
Hap_4	-	A	-----	ATAAT	G	G	T	-----------	TATACAACTTTTCTATA	ACTTTT	C	T	T	C	T	A	A	T	T
Hap_5	-	A	-----	ATAAT	G	G	T	-----------	TATACAACTTTTCTATA	ACTTTT	C	T	G	C	T	A	A	C	G
Hap_6	△	C	-----	ATAAT	G	T	G	-----------	TATACAACTTTTCTATA	ACTTTT	T	C	G	C	A	A	T	T	G
Hap_7	-	A	-----	ATAAT	G	G	T	-----------	TATACAACTTTTCTATA	ACTTTT	C	T	G	C	T	A	A	T	T
Hap_8	-	A	-----	ATAAT	G	G	T	TGTTATATAGA	TATACAACTTTTCTATA	ACTTTT	C	T	G	C	T	A	A	T	T
Hap_9	-	A	-----	ATAAT	G	G	T	-----------	TATACAACTTTTCTATA	ACTTTT	C	T	G	C	T	A	A	T	G
Hap_10	-	A	-----	-----	G	G	T	-----------	TATACAACTTTTCTATA	ACTTTT	C	T	T	C	T	A	A	T	T
Hap_11	-	A	-----	ATAAT	G	G	T	-----------	TATACAACTTTTCTATA	ACTTTT	C	T	T	C	T	A	A	T	G
Hap_12	-	A	-----	ATAAT	G	G	T	-----------	-----------------	------	C	T	G	C	T	A	A	T	G
Hap_13	-	A	TAATAT	ATAAT	G	G	T	-----------	TATACAACTTTTCTATA	ACTTTT	C	T	G	C	T	A	A	T	G

*△: TAAAAATTATAATTATATTAGAATTCGTATATTATACTAAGTATATCTAAATACTAAGTATATCTAAGTGAGTATATATATCTAATAAGTGCAAGGAATGTAGAATTAAATAAAAGGACTTG
