# Supplementary material for: Land masses and oceanic currents drive population structure of Heritiera littoralis, a widespread mangrove in the Indo‐West Pacific
Source: Ecol Evol. 2020 Jun 3;10(14):7349–63. doi: 10.1002/ece3.6460 (PMC7391321; doi:10.1002/ece3.6460)
Supplement: Supplementary file 4 — Appendix S4 [file ECE3-10-7349-s004.pdf]

# Appendix S4

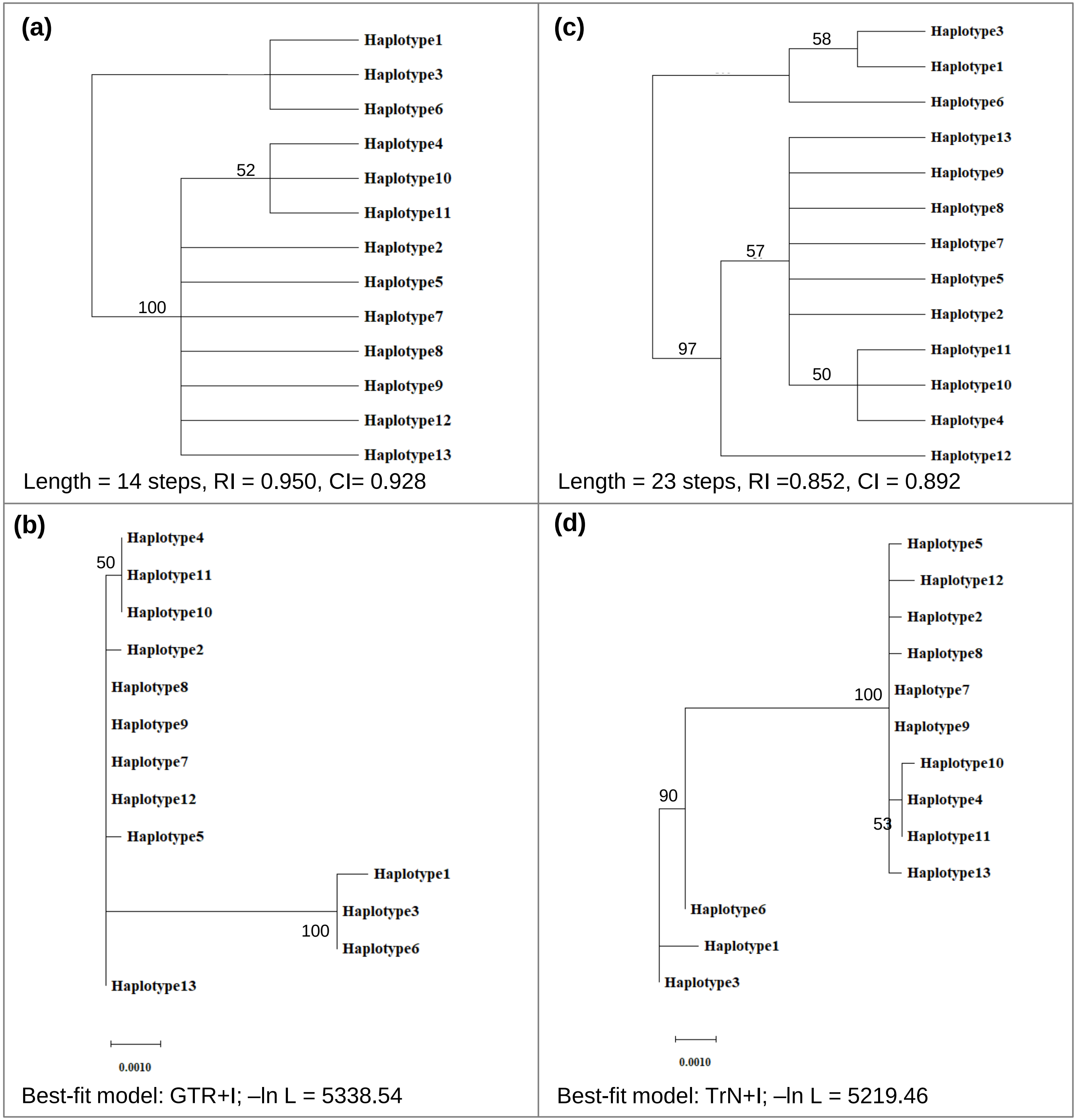


Phylogenetic relationships of 13 haplotypes resolved in *Heritiera littoralis*. The trees are constructed by Maximum-Parsimony (a, c) and Maximum-Likelihood (b, d) methods. Numbers on branches show the supporting ratio obtained from bootstrapping with 1000 replicates. Nodes with supporting ratio less than 50 are collapsed. (a) and (b) are based on the sequence with indels as missing data; (c) and (d) are based on the sequence with indels as single mutation event.
