# Supplementary material for: Land masses and oceanic currents drive population structure of Heritiera littoralis, a widespread mangrove in the Indo‐West Pacific
Source: Ecol Evol. 2020 Jun 3;10(14):7349–63. doi: 10.1002/ece3.6460 (PMC7391321; doi:10.1002/ece3.6460)
Supplement: Supplementary file 5 — Appendix S5 [file ECE3-10-7349-s005.pdf]

Appendix S5

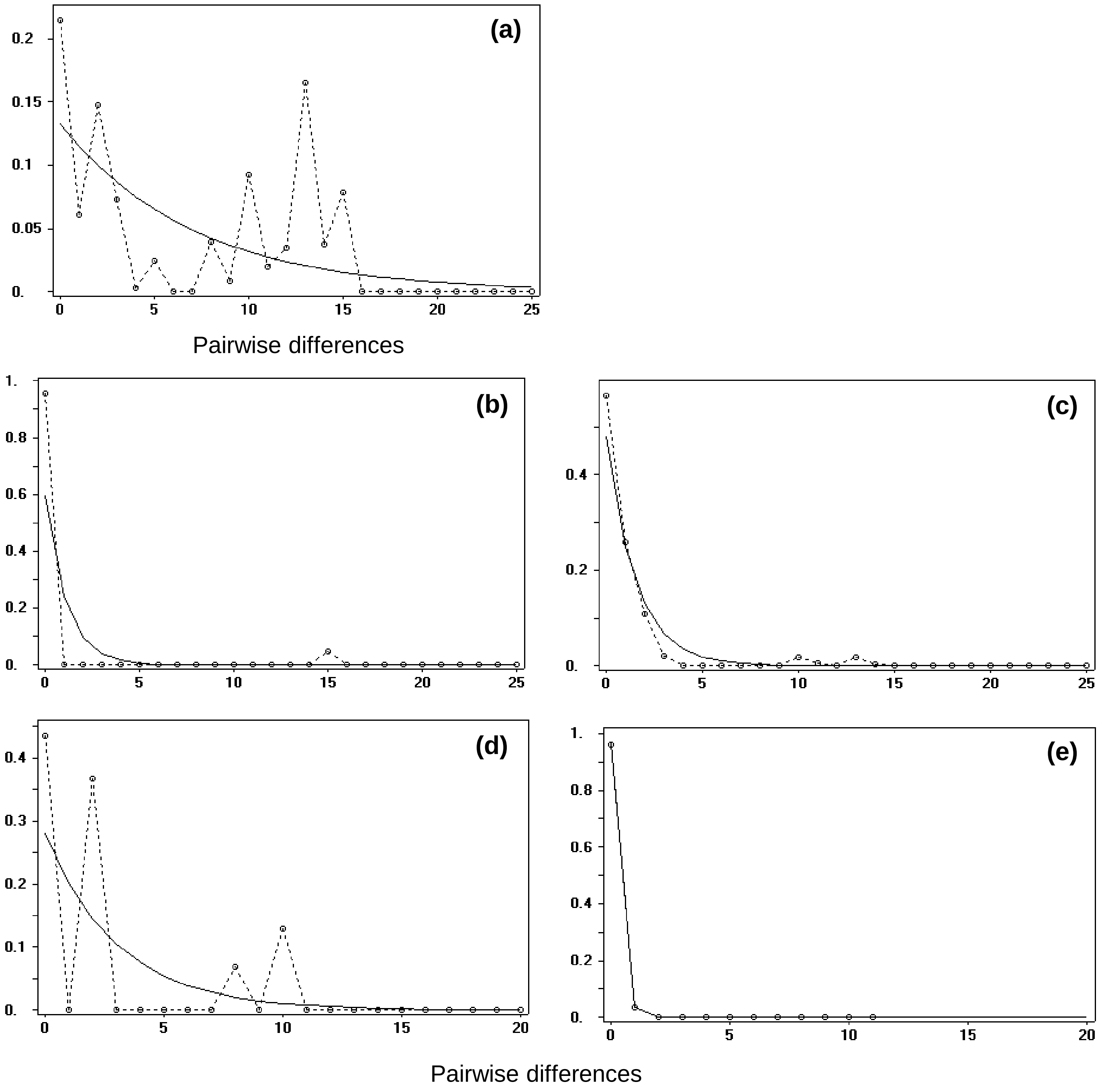

Mismatch distribution analysis for *Heritiera littoralis* – (a) as a whole; (b) for POP I; (c) for POP II; (d) for POP III; (d) for POP IV. The solid lines show observed distributions of pairwise differences among cpDNA haplotypes and the dashed lines represent the distributions expected for an expanding population.
