# Supplementary material for: Land masses and oceanic currents drive population structure of Heritiera littoralis, a widespread mangrove in the Indo‐West Pacific
Source: Ecol Evol. 2020 Jun 3;10(14):7349–63. doi: 10.1002/ece3.6460 (PMC7391321; doi:10.1002/ece3.6460)
Supplement: Supplementary file 6 — Appendix S6 [file ECE3-10-7349-s006.pdf]

Appendix S6: Posterior probability of each of the seven scenarios and their 95% confidence interval based on the logistic estimate by DIYABC; the scenarios are shown in Figure 2

Model	Scenario	Posterior probability	95% Confidence Interval (lower-upper)
ABC1	1	0.086	0.044-0.123
	2	0.0199	0.000-0.063
	3	0.0101	0.000-0.054
	4	0.0045	0.000-0.049
	5	0.3955	0.331-0.459
	6	0.0578	0.013-0.103
	7	0.4263	0.402-0.487
ABC2	1	0.0715	0.000-0.223
	2	0.0598	0.000-0.219
	3	0.5633	0.449-0.678
	4	0.0034	0.000-0.172
	5	0.0113	0.000-0.179
	6	0.1965	0.052-0.341
	7	0.0943	0.000-0.301
ABC3	1	0.0831	0.000-0.185
	2	0.1725	0.081-0.264
	3	0.5571	0.483-0.631
	4	0.0221	0.000-0.186
	5	0.0106	0.000-0.128
	6	0.1368	0.046-0.228
	7	0.0179	0.000-0.133
