# Supplementary material for: Land masses and oceanic currents drive population structure of Heritiera littoralis, a widespread mangrove in the Indo‐West Pacific
Source: Ecol Evol. 2020 Jun 3;10(14):7349–63. doi: 10.1002/ece3.6460 (PMC7391321; doi:10.1002/ece3.6460)
Supplement: Supplementary file 7 — Appendix S7 [file ECE3-10-7349-s007.pdf]

Appendix S7: Numerical results of model checking in terms of one sample and two sample summary statistics for cpDNA loci.

Summary statistics			Observed value			Proportion (simulated<observed)		
			ABC1	ABC2	ABC3	ABC1	ABC2	ABC3
Genic diversities	Proportion of zero values	HP0_1_1	0.083	0.000	0.000	0.060	0.008*	0.031*
		HP0_1_2	1.000	1.000	1.000	0.663	0.834	0.761
		HP0_1_3	0.917	0.364	0.462	0.920	0.065	0.140
	Mean of non-zero values	HM1_1_1	0.045	0.045	0.073	0.013*	0.022*	0.066
		HM1_1_2	0.000	0.000	0.000	0.337	0.166	0.239
		HM1_1_3	0.049	0.391	0.391	0.112	0.927	0.904
	Variance of non-zero values	HV1_1_1	0.000	0.000	0.003	0.005*	0.009*	0.013*
		HV1_1_2	0.000	0.000	0.000	0.444	0.329	0.396
		HV1_1_3	0.000	0.000	0.000	0.089	0.055	0.049*
	Mean of complete distribution	HMO_1_1	0.041	0.045	0.073	0.053	0.088	0.164
		HMO_1_2	0.000	0.000	0.000	0.337	0.166	0.239
		HMO_1_3	0.004	0.249	0.211	0.075	0.996*	0.976*
F_{ST} distances	Proportion of zero values	FP0_1_1&2	0.000	0.000	0.000	0.005*	0.003*	0.003*
		FP0_1_1&3	0.000	0.000	0.231	0.146	0.005*	0.099
		FP0_1_2&3	0.833	0.364	0.308	0.854	0.231	0.075
	Mean of non-zero values	FM1_1_1&2	0.894	0.182	0.162	0.999*	0.394	0.307
		FM1_1_1&3	0.741	0.367	0.567	1.000*	0.986*	1.000*
		FM1_1_2&3	0.983	0.282	0.853	0.985*	0.464	0.981*
	Variance of non-zero values	FV1_1_1&2	0.077	0.151	0.121	0.439	0.797	0.703
		FV1_1_1&3	0.189	0.101	0.145	0.996*	0.955*	0.982*
		FV1_1_2&3	0.001	0.000	0.007	0.070	0.010*	0.036*
	Mean of complete distribution	FMO_1_1&2	0.894	0.182	0.162	1.000*	0.704	0.607
		FMO_1_1&3	0.741	0.367	0.436	1.000*	1.000*	1.000*
		FMO_1_2&3	0.164	0.179	0.590	0.797	0.640	1.000*
Nei's distances	Proportion of zero values	NP0_1_1&2	0.000	0.000	0.000	0.035*	0.059	0.111
		NP0_1_1&3	0.000	0.000	0.000	0.284	0.113	0.153
		NP0_1_2&3	0.833	0.364	0.308	0.906	0.286	0.095
	Mean of non-zero values	NM1_1_1&2	0.897	0.178	0.149	1.000*	0.722	0.638
		NM1_1_1&3	0.732	0.209	0.338	1.000*	0.994*	1.000*
		NM1_1_2&3	0.987	0.056	0.743	0.990*	0.208	0.979*
	Variance of non-zero values	NV1_1_1&2	0.080	0.156	0.130	0.485	0.856	0.820
		NV1_1_1&3	0.195	0.145	0.106	0.993*	0.998*	0.992*
		NV1_1_2&3	0.000	0.000	0.021	0.105	0.056	0.183
	Mean of complete distribution	NMO_1_1&2	0.897	0.178	0.149	1.000*	0.816	0.724
		NMO_1_1&3	0.732	0.209	0.338	1.000*	0.998*	1.000*
		NMO_1_2&3	0.165	0.036	0.514	0.855	0.230	0.999*

* indicates proportions of simulated data having value lower than 5% or greater than 95% of the observed dataset
