# Supplementary material for: Land masses and oceanic currents drive population structure of Heritiera littoralis, a widespread mangrove in the Indo‐West Pacific
Source: Ecol Evol. 2020 Jun 3;10(14):7349–63. doi: 10.1002/ece3.6460 (PMC7391321; doi:10.1002/ece3.6460)
Supplement: Supplementary file 8 — Appendix S8 [file ECE3-10-7349-s008.pdf]

Appendix S8

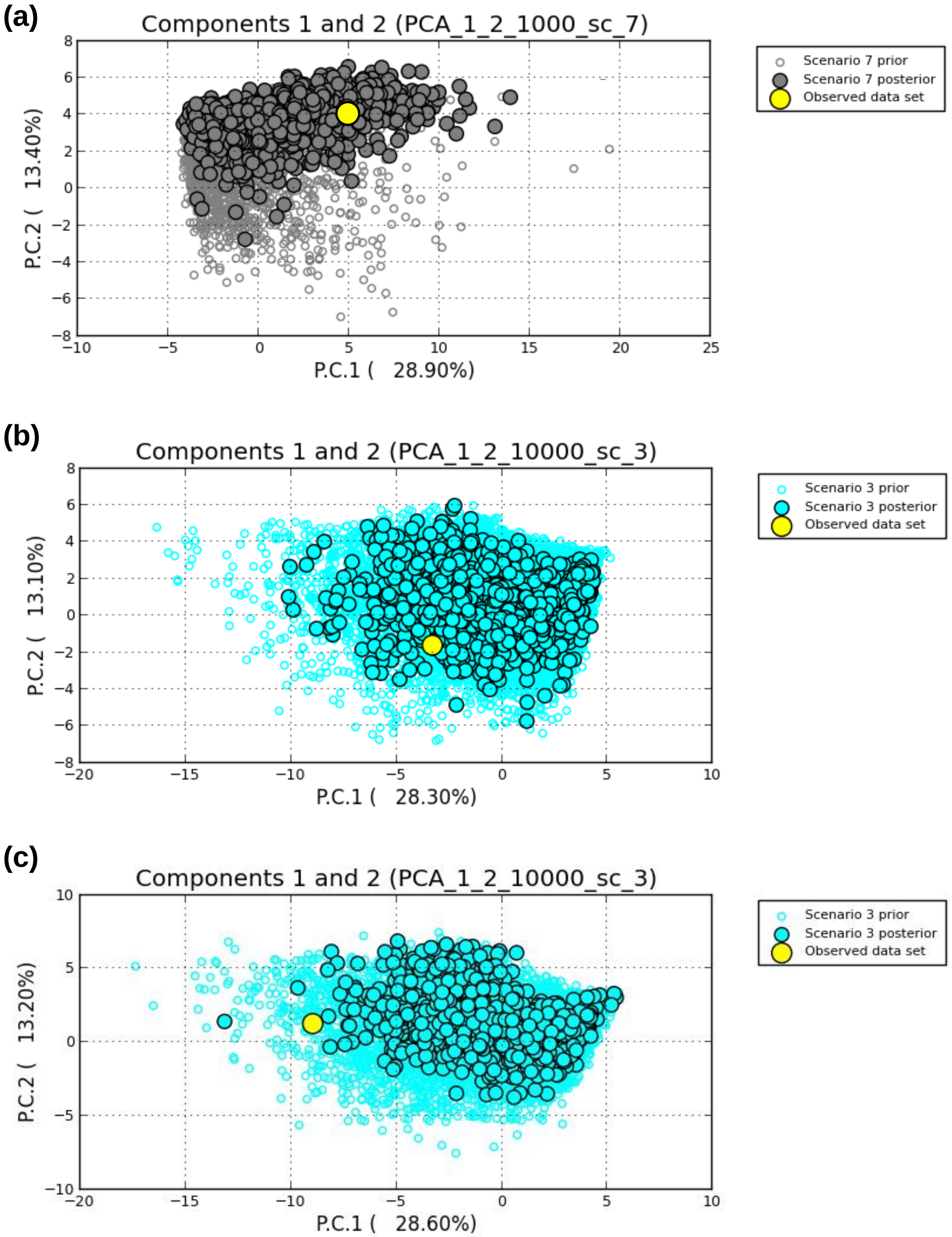

Principal Component Analysis (PCA) of "model checking" for selected scenarios – (a) scenario 7 for ABC1, (b) scenario 3 for ABC2, and (c) scenario 3 for ABC3; as estimated in the software DIYABC ver.2.0.
